# The Use of Knockout Mice Reveals a Synergistic Role of the *Vav1* and *Rasgrf2* Gene Deficiencies in Lymphomagenesis and Metastasis

**DOI:** 10.1371/journal.pone.0008229

**Published:** 2009-12-14

**Authors:** Sergio Ruiz, Eugenio Santos, Xosé R. Bustelo

**Affiliations:** Centro de Investigación del Cáncer and Instituto de Biología Molecular y Celular del Cáncer, Consejo Superior de Investigaciones Científicas (CSIC)–University of Salamanca, Salamanca, Spain; Universidade Federal do Rio de Janeiro (UFRJ), Brazil

## Abstract

**Background:**

Vav1 and RasGRF2 are GDP/GTP exchange factors for Ras superfamily GTPases with roles in the development and/or effector functions of T–lymphocytes.

**Methodology/Principal Findings:**

Given that the phenotype of *Vav1* ^–/–^, *Rasgrf2* ^–/–^ and *Vav1* ^–/–^;*Rasgrf2* ^–/–^ mice has been studied so far in young animals, we decided to explore the long–term consequences of the inactivation of those loci in the immune system. Unexpectedly, our studies revealed that the inactivation of the *Vav1* proto–oncogene favors the formation of lymphoblastic lymphoma–like tumors in aging mice. Those tumors, that can be found either localized exclusively inside the thymus or widely disseminated in hematopoietic and non–hematopoietic tissues, are composed of CD3^+^ lymphoblasts that display heterogeneous combinations of CD4 and CD8 surface markers. Interestingly, the additional deletion of the *Rasgrf2* gene induces a shortening in the latency period for the development of those tumors, an increase in the percentage of disseminated tumors outside the thymus and, as a result, higher mortality rates.

**Conclusions/Significance:**

These data reveal unexpected negative roles for Vav1 and RasGRF2 in different stages of T–cell lymphoma progression. They also suggest that the inactivation of Vav1 function may represent an inadequate strategy to treat T–cell lymphomas, especially those associated with low levels of *Rasgrf2* gene expression.

## Introduction

Ras and Rho/Rac proteins play essential roles in normal signal transduction and pathological states, since they activate intracellular pathways that impinge directly in biological processes related to cell proliferation, survival and motility [1]-[6]. Under normal conditions, these proteins cycle between an inactive, GDP–bound state and an active, GTP–bound conformation. The cycling between these two conformations is regulated by GDP/GTP exchange factors (GEFs), GTPase activating proteins (GAPs) and, in some cases, by Rho GDP dissociation inhibitors (RhoGDIs). GEFs promote the rapid exchange of GDP by GTP during cell signaling, thereby helping the rapid transition of Ras and Rho/Rac GTPases from the inactive to active states [7]. GAP proteins enhance the hydrolysis rates of bound GTP molecules, thus favoring the inactivation of Ras and Rho/Rac GTPases at the end of the stimulation cycle [7]. Finally, RhoGDIs contribute to the downmodulation of Rho/Rac–dependent GTPase pathways by retrieving the GTPases from membranes and, subsequently, by maintaining them sequestered in the cytosol in their inactive, GDP–bound conformation [8]-[10]. The importance of this regulatory cycle is underscored by the observation that point mutations affecting either GTP hydrolysis or the intrinsic GDP/GTP exchange of Ras and Rho/Rac proteins lead to the generation of GTPases with high (in the case of Ras GTPases) or intermediate (in the case of Rho/Rac proteins) oncogenic potential [1], [5], [6].

Whereas it has been always assumed that tumorigenic processes could be positively and negatively regulated by GEFs and GAPs/RhoGDIs, respectively, recent data have revealed that this regulatory picture is more complex than initially anticipated. For example, it has been recently shown that RhoGDIs are required for the efficient transforming activity of the GTPase Cdc42, an observation that suggest that these GTPase inhibitors may also play positive roles in the translocation and/or effector phase of these GTPases [11]. On the other hand, recent observations have shown that *RASGRF2*, a human gene encoding a dual GEF with specificity for Ras and Rho/Rac proteins, is either hypermethylated or downregulated transcriptionally in cancer cell lines and primary tumors [12]-[15], a biological property reminiscent of genes associated with tumor suppressor activities. Consistent with this possibility, it has been recently demonstrated that the overexpression of RasGRF2 affects negatively the transforming properties of a colon cancer cell line [15]. Despite the above results, there is no actual evidence indicating that this GEF could play tumor suppressor–like activities in tumorigenic processes in vivo. Likewise, we have no information regarding the implication of other GEFs in the negative regulation of tumor growth. In fact, the overexpression of wild type or the expression of constitutively–active mutant versions of many GEFs have been usually associated to oncogenesis rather than to growth inhibitory signals. For example, the transforming activity of the human *VAV1* and the *RASGRP1* oncogenes, two loci encoding GEFs for either Rho/Rac (*VAV1*) or Ras (*RASGRP1*), was the biological read–out that allowed the discovery of these important regulatory proteins [16], [17].

Recently, we described that the Vav1 and RasGRF2 GEFs play synergistic roles in the activation of specific T–cell receptor (TCR) downstream signaling pathways, including the optimal activation of phospholipase C–γ, the stimulation of the nuclear factor of activated T–cells, and the transcriptional upregulation of cytokine genes [18]. Consistent with this signaling cross–talk, we demonstrated that the deletion of the mouse *Rasgrf2* gene aggravated the defective proliferative responses of mature, TCR–stimulated *Vav1* ^–/–^ T–cells [18]. Given the aforementioned defects, we decided to perform long–term studies with *Vav1* ^–/–^, *Rasgrf2* ^–/–^, *Vav1* ^–/–^;*Rasgrf2* ^–/–^ and control animals to investigate the possible development of additional, age–dependent immune defects such as autoimmune disease. Unexpectedly, we found that the loss of *Vav1* and *Rasgrf2* genes cooperated synergistically in the development of very aggressive T–cell lymphomas in mice. These observations indicate that in some specific signaling contexts, the absence of GEF function may contribute to, rather than impacting negatively on, tumorigenesis. They also suggest that anti–cancer therapies directed against GEFs may not be advisable in some tumor types.

## Results

### Synergistic Effect of *Vav1* and *Rasgrf2* Gene Deficiencies in Leukemia/Lymphoma Development

**Figure 1:**
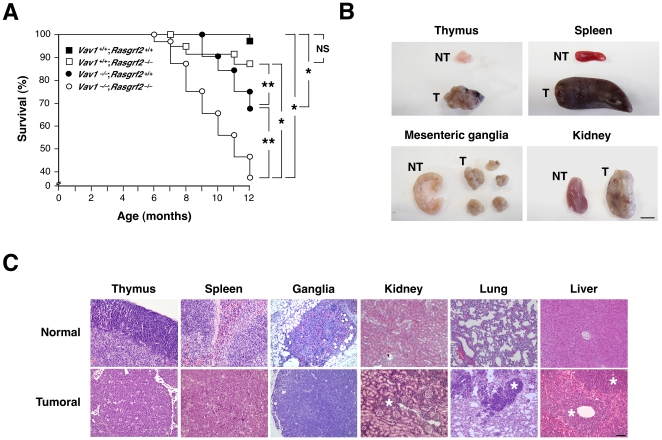
The combined *Vav1/Rasgrf2* gene deficiency enhances the rates of lymphomas. (**A**) Kaplan–Meyer distribution graph showing the percentage of survival rates of mice of the indicated genotypes. NS, non–statistically significant values; *, *P*<0.01; **, *P*<0.05. (**B**) Examples of non–tumorigenic (NT) and tumorigenic (T) tissues obtained from two *Vav1* ^–/–^;*Rasgrf2* ^–/–^ mice. Scale bar, 1 cm. (**C**) Hematoxylin/eosin stained sections of healthy and tumoral tissues obtained from a healthy wild type (upper panel) and a tumor–bearing *Vav1* ^–/–^;*Rasgrf2* ^–/–^ (lower panel) mouse, respectively. Scale bar, 100 µm. Asterisks, tumoral cells infiltrates in non–hematopoietic tissues.

Given the defects observed in *Vav1* ^–/–^, *Rasgrf2* ^–/–^ and *Vav1^–/–^*;*Rasgrf2* ^–/–^ mice in TCR–mediated responses, we decided to perform long–term studies with those animals to investigate the possible development of additional, age–dependent immune defects. To avoid interferences with genetic polymorphisms, all mouse strains were previously homogenized in the B10.BR genetic background. We observed that whereas wild type (6%, *n* = 32) and *Rasgrf2* ^–/–^ (12%, *n* = 24) animals showed a low disease/death rate up to a year of age, *Vav1^–/–^* animals (32%, *n* = 25) and, to a larger extent, the double *Vav1* ^–/–^;*Rasgrf2* ^–/–^ mice (68.6%, *n* = 42) were prone to either dying prematurely or falling sick as they aged (**Figure 1A**). The latency periods for disease development were also shorter in *Vav1* ^–/–^;*Rasgrf2* ^–/–^ mice than in the rest of genotypes (**Figure 1A**). Unexpectedly, the examination of dead and euthanized sick animals indicated that the disease/death cause was the presence of highly disseminated lymphoid tumors rather than any type of autoimmune disease. Specifically, we observed hyperplasia of thymi, massive splenomegalies and lymphadenopathies in most of the animals analyzed (**Figure 1B**). The above tissues also showed a disruption of their normal histological structures as a consequence of the extensive growth and/or colonization of the whole tissue by tumor cells. Due to this, the typical separation of cortical and medullar areas, the white and red pulp areas, and the germinal/peripheral centers where completely blurred or eliminated in the thymi, spleen and mesenteric lymph nodes, respectively (**Figure 1C**, compare upper and lower panels). We also detected extensive infiltrations of tumor cells in kidneys (**Figure 1C**), lungs (**Figure 1C**), liver (**Figure 1C**), intestine (data not shown), and bone marrow (see below). Interestingly, we did not find increased rates of leukemia/lymphoma in aging *Vav2* ^–/–^, *Vav3* ^–/–^ or *Vav2* ^–/–^;*Vav3* ^–/–^ mice when compared to control littermates (*n*>30 for each genotype, data not shown). These results indicated that the *Vav1* proto–oncogene deficiency in mice leads in the long–term to leukemia/lymphoma and that the loss of the *Rasgrf2* gene further accentuates the progression of that disease.

### Characterization of Tumors Present in *Vav1* ^–/–^;*Rasgrf2* ^–/–^ Mice

**Figure 2:**
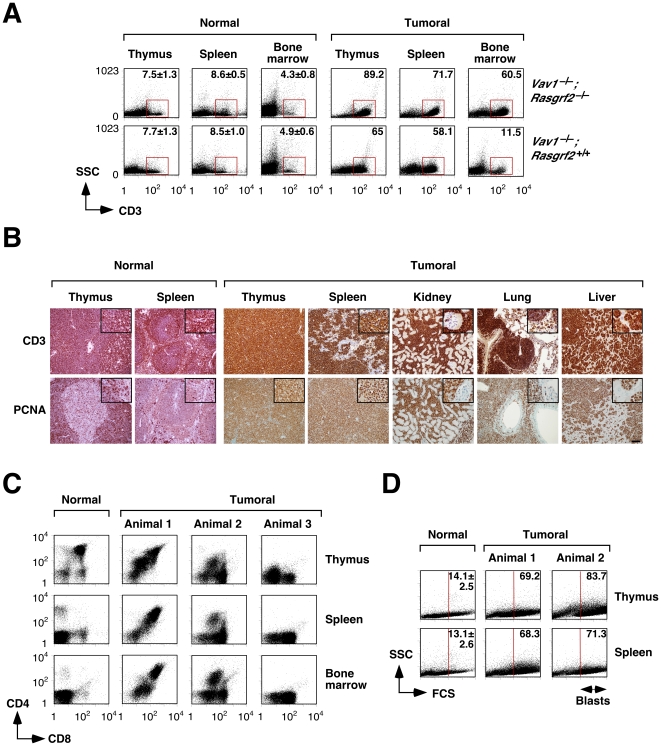
Characterization of lymphoid tumors. (**A**) Cell suspensions from healthy and tumoral tissues obtained from mice of the indicated genotypes (right) were stained with anti–CD3 antibodies and subjected to flow cytometry analysis. Red squares highlight CD3^+^ positive cell populations present in the animals under study. The percentage of the CD3^+^ population in each case is indicated inside each panel. SSC, side scatter. (**B**) Tissues from a tumor–bearing *Vav1* ^–/–^;*Rasgrf2* ^–/–^ mouse (panels 3–7 counting from the left) were stained with either anti–CD3 (top panels) or anti–PCNA (bottom panels) using immunohistochemistry techniques. As control, we stained thymus and spleen sections obtained from a wild type mouse (first and second panel from left, respectively). Scale bar, 100 µm. (**C**) Cell suspensions were isolated from a healthy and three tumor–bearing *Vav1* ^–/–^;*Rasgrf2* ^–/–^ animals, stained with anti–CD4 and anti–CD8 antibodies, and analyzed by flow cytometry. (**D**) Cell suspensions derived from the indicated tissues of a healthy and two tumoral *Vav1* ^–/–^;*Rasgrf2* ^–/–^ mice were collected and blasts visualized by flow cytometry. In the *y* and *x* axes, the SSC and FCS values range from 0 to 1,024, respectively. The vertical lines indicate the FCS point from where gated cells were considered as blasts. FCS, forward scatter. The percentage of blasts in each case is indicated inside each panel. *n* = 5 and 10 for healthy and tumor–bearing animals, respectively (panels A–D).

Flow cytometry analysis of cells obtained from the thymus, spleen and bone marrow of tumor–bearing *Vav1* ^–/–^;*Rasgrf2* ^–/–^ and *Vav1* ^–/–^ animals revealed the presence of a new population characterized by the surface expression of CD3, a T–cell specific marker (**Figure 2A**). The percentage of cells belonging to this population was highly variable among the animals under study (12–90%, see an example by comparing the left panels of **Figure 2A**). Consistent with these cytometry data, immunohistochemical analysis of tissue sections indicated that the tumor cells present in both hematopoietic and non–hematopoietic tissues displayed high levels of CD3 expression (**Figure 2B**). By contrast, these tumor cells were more heterogeneous in terms of the surface expression of the CD4 and CD8 markers. Thus, we found cases in which tumor cells were homogeneously double positive for those markers (**Figure 2C**, animal #1), cases in which the leukemia/lymphomas were composed of two separate CD4^+^CD8^+^ and CD8^+^ populations (**Figure 2C**, animal #2), and cases in which single CD8^+^ cells were observed in the tumor cell population (**Figure 2C**, animal #3). Although the distributions of the CD4 and CD8 markers were rather stochastic in the tumor cells analyzed, we did find that single positive T–cells were usually skewed towards the CD8^+^ rather than to the CD4^+^ lineage. Despite this variability among animals, the surface immunophenotype of the leukemia/lymphoma cells from the same animal was similar independently of the tissue source they were obtained from (**Figure 2C**).

We next investigated the proliferative status of the leukemia/lymphoma cells present in these animals. Flow cytometry analyses indicated that tumor cells contained large percentages of blasts, an indication of high proliferative rates (**Figure 2D**). Immunohistochemistry experiments confirmed these results, since all tumors sections analyzed displayed higher expression levels of the proliferating cell nuclear antigen (PCNA), a processivity factor for DNA polymerase δ whose expression correlates with active cell proliferation (**Figure 2B**) [19]. Despite this, we could not observe any increase in the expression of the surface marker CD69 in thymic, splenic, or bone–marrow derived leukemia/lymphoma cells by flow cytometry (data not shown). Since CD69 is upregulated in proliferating T–cells in a TCR–dependent manner [20], these results suggest that the proliferation of those leukemia/lymphoma cells occurs independently of TCR engagement. Similar data were obtained with tumors derived from single *Vav1* ^–/–^, *Rasgrf2* ^–/–^ and wild type animals.

### Thymic Origin of Tumors

**Figure 3:**
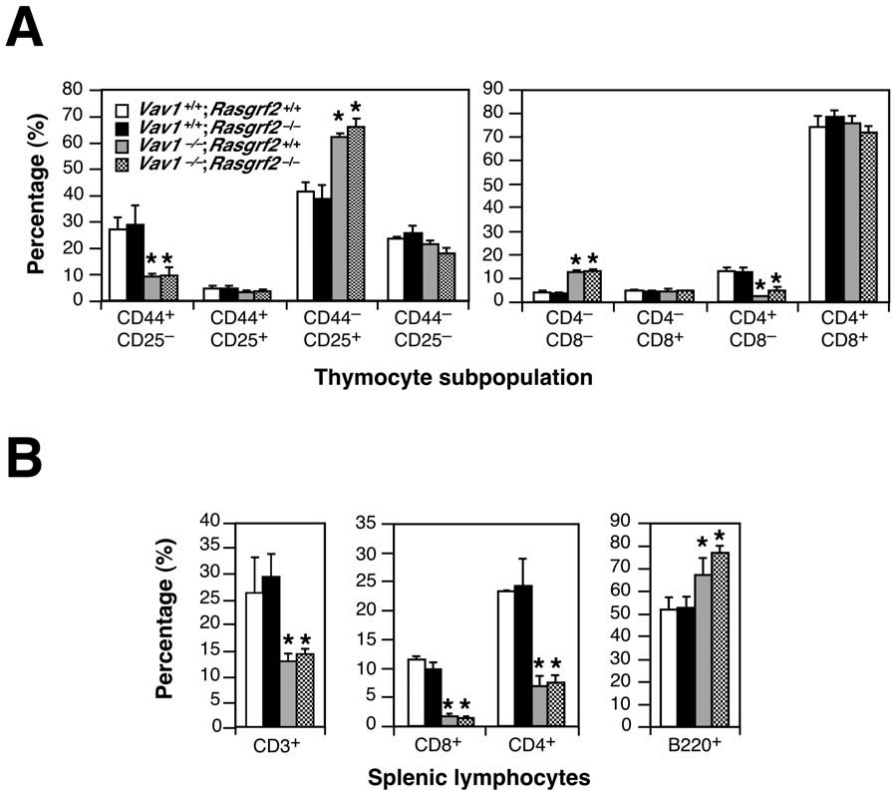
The *Rasgrf2* gene deficiency does not affect the normal development of T–cells (A) or mature lymphocyte (B) cell numbers. Bar graphs show the mean percentages of thymocyte (upper panels) and splenic lymphocyte (lower panels) subpopulations derived from animals of the indicated genotypes. Error bars represent the standard deviation (*n* = 5). *, *P*<0.01 compared to values obtained with wild type controls.

In order to investigate the origin and ontology of those tumor cells, we decided to study the T–cell compartment in tumor–free animals. To this end, we performed histological and flow cytometry analyses of wild type, *Vav1* ^–/–^, *Rasgrf2* ^–/–^ and *Vav1* ^–/–^;*Rasgrf2* ^–/–^ animals that were six– to eight–week–old, an age period in which we had not detected any deaths or sickness sign in the animals. In addition, we focused our attention on mice that had survived up to a year of age and that, therefore, were categorized as “disease–free” animals in our first screening phase. In the case of young mice, we observed no obvious tumoral or pre–neoplasic manifestations in any of the animals analyzed (*n* = 20). Moreover, we found that wild type and *Rasgrf2* ^–/–^ mice exhibited the expected distribution of thymocyte subpopulations (**Figure 3A**) and of splenic CD3^+^, CD4^+^ and CD8^+^ T–cells (**Figure 3B**). As expected [21], *Vav1* ^–/–^ mice did show alterations in the percentage of thymocyte subpopulations due to a defective transition of CD4^–^CD8^–^ cells from the CD44^–^CD25^+^ to the CD44^–^CD25^–^ stage (**Figure 3A**). Although the relative percentages of CD4^+^CD8^+^ thymocytes did not change when compared with wild type counterparts and *Rasgrf2* ^–/–^ mice (**Figure 3A**), we did observe a reduction in the total numbers of production of these cells that resulted in lower levels of thymic cellularity (data not shown). This defect has been previously attributed to a defective positive selection that leads to enhanced mortality rates in this thymic subpopulation [21]. As previously reported [21], [22], *Vav1* ^–/–^ mice also manifested T–lymphopenia as inferred by the increased in the percentage of B220^+^ B–cells in the spleen (**Figure 3B**). The combined inactivation of the *Rasgrf2* and *Vav1* loci did not aggravate the thymocyte developmental/selection defects or the T–cell lymphopenia induced by the single *Vav1* gene deficiency (**Figure 3**). Hence, these results indicate that young animals do not have neoplasic or pre–neoplasic manifestations and that the inactivation of the *Rasgrf2* gene does not accentuate the already severe immune defects caused by the Vav1–deficiency.

**Figure 4:**
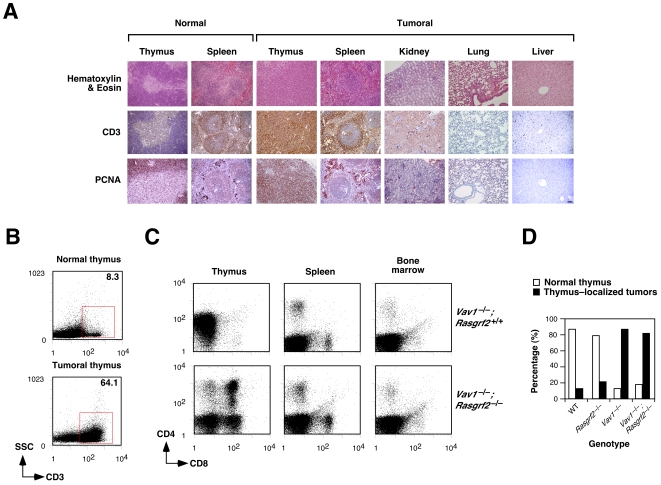
The lymphomas found in *Vav1^–/–^*;*Rasgrf2* ^–/–^ and *Vav1* ^–/–^ derive from the thymus. (**A**) Hematoxylin/eosin, anti–CD3 and anti–PCNA staining of tissue sections obtained from a *Vav1* ^–/–^;*Rasgrf2* ^–/–^ mouse with a thymus–localized lymphoma (panels 3–7 counting from left). As control, we stained thymus and spleen sections obtained from a wild type mouse (first and second panel from left, respectively). Scale bar, 100 µm. (**B**) Cell suspensions were obtained from a healthy *Vav1* ^–/–^;*Rasgrf2* ^–/–^ (top) and a *Vav1* ^–/–^;*Rasgrf2* ^–/–^ mouse with a thymus–localized tumor (bottom), stained with anti–CD3 antibodies and analyzed by flow cytometry. The red square highlights the abnormal, CD3^+^ positive cell population found in the thymus–localized tumor. The percentage of CD3^+^ cells in each case is indicated inside each panel. (**C**) Cell suspensions were obtained from a *Vav1* ^–/–^;*Rasgrf2* ^+/+^ (top) and a *Vav1* ^–/–^;*Rasgrf2* ^–/–^ (bottom) mouse containing thymus–localized tumors, stained with anti–CD4 and anti–CD8 antibodies and analyzed by flow cytometry. Results similar to those shown in panels A–C were observed in 5 independent determinations. (**D**) Graph showing the percentage of one–year old mice of the indicated genotypes with thymus–localized tumors.

In contrast to the results with young animals, we observed that one–year–old, “tumor–free” animals displayed a high frequency of hyperplasic thymi with disrupted compartmentalization in cortical and medullary regions (**Figure 4A**; data not shown). As in the case of leukemia/lymphoma cells from the aforementioned tumor–bearing animals, these thymocytes expressed excess amounts of CD3^+^ (**Figure 4A,B**), were PCNA positive (**Figure 4A**), and showed a heterogeneous display of CD4 and CD8 surface molecules (**Figure 4C** and data not shown). However, we could not find any infiltration of CD3^+^ lymphoma cells in other tissues (**Figure 4A,C**). Furthermore, no signs of splenomegalia or lymphadenopathy could be detected in these “tumor–free” animals (data not shown). Kidneys, lungs and livers were also normal in these mice (**Figure 4A**). These thymic alterations were detected at very high rates in animals of both *Vav1* ^–/–^ (≈85%, *n* = 14) and *Vav1* ^–/–^;*Rasgrf2* ^–/–^ (≈80%, *n* = 14) genotypes (**Figure 4D**). These results indicate that the T–cell leukemia/lymphomas found in these animals are of thymic origin. As in the case of invasive/metastatic leukemia/lymphomas, this thymus–restricted tumorigenesis was observed, although at lower rates, in one–year–old wild type (≈17%, *n* = 25) and *Rasgrf2* ^–/–^ (≈22%, *n* = 20) mice (**Figure 4D**), further suggesting that Vav1 accentuates the intrinsic tendency of this mouse strain to develop thymic lymphomas rather than promoting tumorigenesis in an autonomous fashion.

### The Expression of RasGRF2 and Vav1 Is Altered in Very Restricted Patients Subgroups Affected by Hematopoietic Malignancies

**Figure 5:**
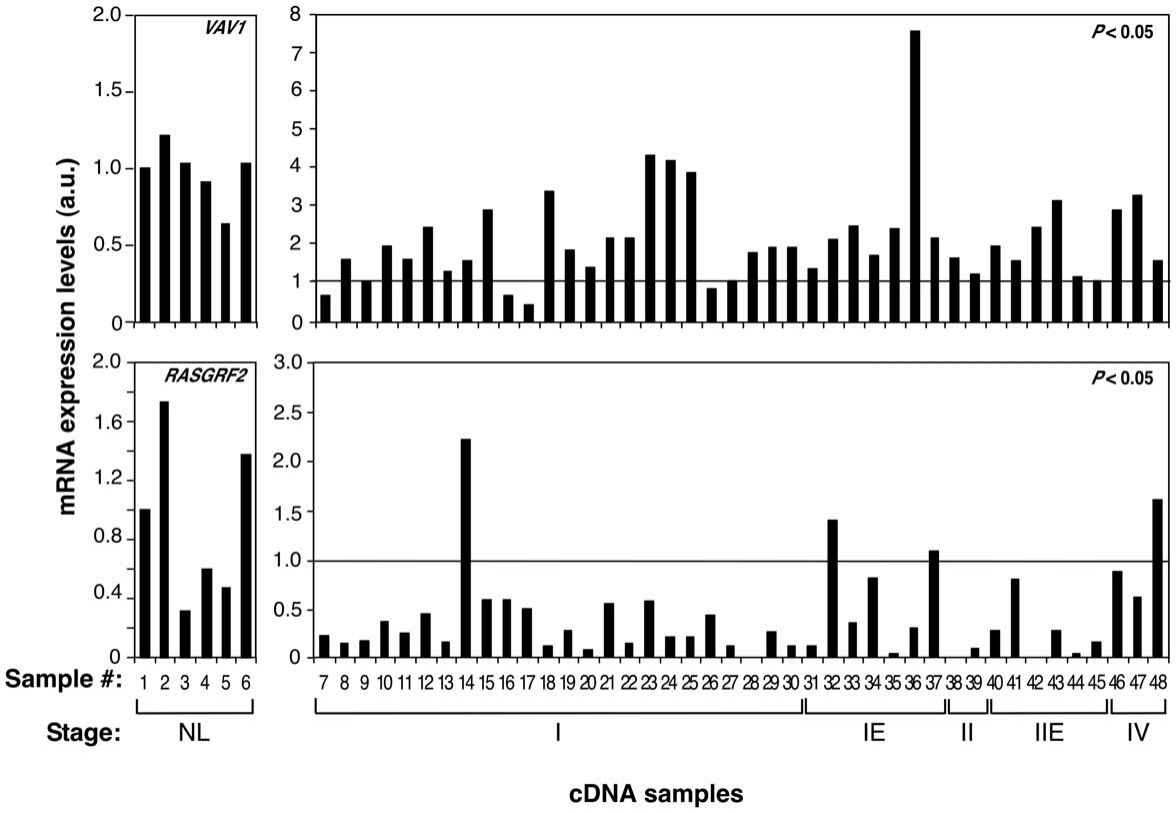
Human *RASGRF2* transcript levels are downregulated in some malignant hemopaties when compared to normal lymphocytes. *Left panels*, cDNA samples from six control lymphocyte samples were subjected to quantitative RT–PCR analysis to observe the variations in basal expression levels of *VAV1* (top panel) and *RASGRF2* (bottom panel) genes and to obtain the average expression values for each gene (which were given an arbitrary value of 1) to be used as reference in the subsequent RT–PCR experiments with tumor samples. *Right panels*, expression levels of *VAV1* (top panel) and *RASGRF2* (lower panel) mRNAs in lymphoma/leukemias (samples 7–48) of the indicated clinical stages by RT–PCR. The horizontal lane represents the mean value of the control samples. *P* values in this figure were estimated by comparing the groups of healthy and tumor samples. a.u., arbitrary units.

**Figure 6:**
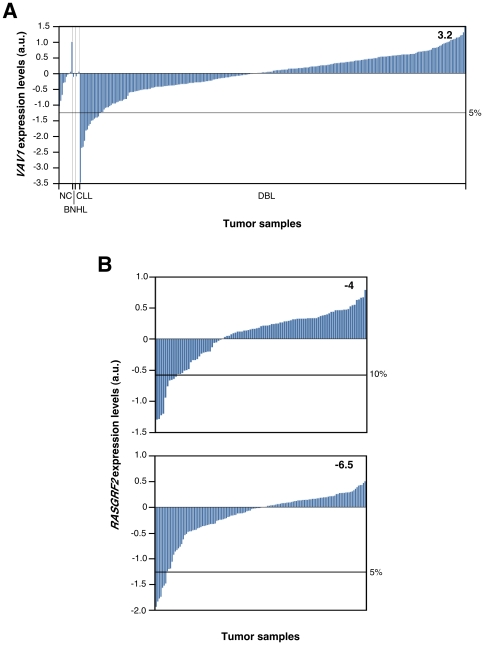
Examples of patient subgroups showing reduced expression levels of *VAV1* (A) and *RASGRF2* (B) genes. Graphs show the variation in expression of those genes in samples obtained from patients in non–characterized tumors (NC) (**A**), B–cell non–Hodgkin’s lymphoma (BNHL) (**A**), chronic lymphocytic leukemia (CLL) (**B**), diffuse large B–cell lymphoma (DBL) (**A**), B–cell acute lymphoblastic leukemia (**B**, upper panel) and acute lymphoblastic leukemia (**B**, lower panel). Outlier groups were identified as indicated in Materials and Methods. The respective CODA scores of each hit are indicated within each panel. The percentile of tumors showing the significant outlier values for the indicated gene is indicated on the right. See **Tables 2** and **3** for further details. a.u., arbitrary units.

The above observations indicated that the deletion of the *Vav1* and/or *Rasgrf2* gene could play a role in the development of lymphoid tumors in humans. To get some insight into this possibility, we first resorted to a quantitative reverse transcription (RT)–PCR strategy to investigate the expression levels of both transcripts in a commercial cDNA collection containing samples from lymphocytes of healthy individuals and 42 independent samples from patients affected by lymphoma/leukemia. Those samples included several stages of lymphoma progression as well as a number of B–cell– and T–cell–derived tumors. We did not find any clear reduction in the *VAV1* mRNA levels in human lymphoma samples when compared with those present in lymphocytes from healthy individuals (**Figure 5**, upper panel). On the contrary, we recorded a 30% of cases in which *VAV1* mRNA levels were at least 2–fold higher than those found in normal lymphocytes. Instead, we detected a reduction in *RASGRF2* transcript levels in 81% of all the human lymphoma cases analyzed (**Figure 5**, lower panel). Total elimination of the mRNAs for this protein was observed in a small lymphocytic lymphoma (stage I, sample 28), a peripheral T–cell lymphoma (stage II, sample 38), and a diffuse large B–cell lymphoma (stage IIE, sample 42) (**Figure 5**, lower panel). No statistically significant upregulated levels of the *RASGRF2* mRNA were observed in these samples (**Figure 5**, lower panel). Since the number of samples available for the RT–PCR experiment was rather limited, we decided to expand these studies using in silico screenings with the microarray data present in the Oncomine site (www.oncomine.org). To this end, we first used a standard bioinformatics comparison tool available in that database to investigate whether the expression of *VAV1* and/or *RASGRF2* genes was deregulated in hematopoietic tumor cells relative to their normal cell counterparts. Since few data are available on T–cell lymphomas, we used microarray data derived from studies conducted on leukemia, lymphoma and myeloma samples (see Materials and Methods). These analyses indicated that *VAV1* mRNA was overexpressed in chronic lymphocytic leukemia, diffuse large B–cell lymphoma and smoldering myeloma relative to control cells (**Table 1**). Instead, reduced levels of this transcript were observed in another chronic lymphocytic leukemia cohort and in hairy cell leukemia (**Table 1**). In contrast to the RT–PCR data, we found no significant variations in the expression levels of the *RASGRF2* mRNA in the microarray data available at the Oncomine site, indicating that this gene is not commonly deregulated at least in the case of the hematopoietic tumors selected in this study. We next analyzed whether the expression levels of *VAV1* and *RASGRF2* genes were significantly altered in specific subgroups of patients affected of leukemia, lymphoma or myeloma. To this end, we used the cancer outlier profile analysis (COPA) method available at the Oncomine database (see Materials and Methods). This analysis allows the identification of small fraction of samples showing statistically significant variations in gene expression from the rest of the population patient cohort, thus bypassing the usual problem of the intrinsic heterogeneity present in all analyses based on clinically–derived tumors [23]. In the case of the *VAV1* mRNA, we found a significant downmodulation in a small subgroup of samples derived from patients affected by diffuse large B–cell lymphoma (**Figure 6A**, **Table 2**), chronic lymphocytic leukemia (**Table 2**), and acute myeloid leukemia (**Table 2**). Instead, we could not detect any patient tumor subgroup showing upregulation of *VAV1* relative to the total patient cohort. In the case of *RASGRF2*, we found groups of patients showing downmodulation of that gene in acute lymphoblastic leukemia (**Figure 6B**, **Table 3**). Upregulation was found in some cases of diffuse large B–cell lymphoma, acute myeloid leukemia and chronic myelogenous leukemia (**Table 3**). These results suggest that the changes in the expression profile of *VAV1* and *RASGRF2* genes are not a general event associated to the progression of hematopoietic tumors. Instead, such variation appears to be concentrated on small, although statistically significant patient subgroups.

**Table 1:** Variation in the expression levels of the *VAV1* mRNA between normal and tumor cells.

Type of Regulation	Type of Tumor and Normal Cell	Gene Rank (top %)	*P* value	Reference
**Upregulation**	Chronic lymphocytic leukemia vs B–cells, CD4^+^ T–cells, germinal center B–cells memory B–lymphocyte and umbilical cord B– and T–cells	3	2.6×10^–6^	[39]
	Chronic lymphocytic leukemia vs B–lymphocytes	9	6.5×10^–4^	[40]
	Diffuse large B–cell lymphoma vsB–cells, CD4^+^ T–cells, germinal center B–cells, memory B–lymphocyte and umbilical cord B– and T–cells	8	6.8×10^–4^	[39]
	Smoldering myeloma vs bone marrow cells	5	2.8×10^–6^	[41]
**Downregulation**	Chronic lymphocytic leukemia vs B–cells, centroblasts, memory B–cells, naïve pregerminal center B–cells and small cleaved follicle center cells	3	1.4×10^–9^	[42]
	Hairy cell leukemia vs B–cells, centroblasts, memory B–cells, naïve pregerminalcenter B–cells and small cleaved follicle center cells	9	2.4×10^–6^	[42]

* Searches were done using the following Oncomine parameters: gene: *VAV1* or *RASGRF2*; analysis type: cancer vs normal analysis; dataset sizes: microarray data with a minimum of 75 independent samples; microarray platform: non restricted; gene rank, top 10%; *P* value≤0.001.

**Table 2:** Outlier analysis of the expression of the *VAV1* mRNA in hematopoietic tumors.

Type of Regulation	Tumor type	Gene Rank (top %)	COPA value	Percentile (%)	Reference
**Downregulation**	Diffuse large B–cell lymphoma	8	–3.210	5	[43]
	Chronic lymphocytic leukemia	8	–5.073	5	[40]
	Acute myeloid leukemia	4	–3.539	5	[44]

* Searches were done using the following Oncomine parameters: gene: *VAV1*; analysis type: outlier analysis; cancer type: leukemia, lymphoma and myeloma; dataset sizes: microarray data with a minimum of 75 independent samples; microarray platform: non restricted; gene rank, top 10%; *P* value≤1×10^–4^; COPA value: less than –3.

**Table 3:** Outlier analysis of the expression of the *RASGRF2* mRNA in hematopoietic tumors.

Type of Regulation	Tumor type	Gene Rank (top %)	COPA value	Percentile (%)	Reference
**Upregulation**	Diffuse large B–cell lymphoma	10	3.169	5	[45]
	Acute myeloid leukemia	5	3.697	10	[46]
	Chronic lymphocytic leukemia	9	3.612	10	[47]
**Downregulation**	Acute lymphoblastic leukemia	3	–6.534	5	[48]
	B–cell acute lymphoblastic leukemia	10	–3.997	10	[49]

* Searches were done using the following Oncomine parameters: gene: *VAV1*; analysis type: outlier analysis; cancer type: leukemia, lymphoma and myeloma; dataset sizes: microarray data with a minimum of 75 independent samples; microarray platform: non restricted; gene rank, top 10%; *P* value≤1×10^–4^; COPA value: less than –3 for downregulated genes and higher than 3 for upregulated genes.

## Discussion

We have shown in this report that the double *Vav1/Rasgrf2* gene deficiency favors the development and progression of lymphoblastic lymphoma–like tumors in mice. These tumors could be classified in two different subsets in function of their level of dissemination within the affected animal. A first subset was characterized by its exclusive intrathymic localization. The second subset showed that localization and, in addition, massive infiltrations in extra–thymic hematopoietic–tissues and non–hematopoietic tissues. This latter tumor subset was more aggressive in nature, leading to elevated mortality rates in the affected mice. Notwithstanding these differences in localization and lethality, we observed that these two tumor subsets shared many common biological features, including the expression of CD3 molecules, the heteroclite expression of CD4 and CD8 surface markers, the upregulation of proteins generally involved in proliferation (PCNA) and the lack of expression of TCR–induced markers (i.e., CD69). It is likely therefore that these two pathological subsets correspond to two different stages in lymphoma progression. For the sake of simplicity, we will refer to these two populations hereafter as “thymus–localized” and “widespread” lymphomas.

The *Vav1* and *Rasgrf2* gene deficiencies seem to contribute differently to these two lymphoma stages. Thus, the loss of the *Vav1* proto–oncogene increases preferentially the frequency of “thymus–localized” lymphomas in one–year–old animals and, to a much lower extent, the percentage of “widespread” tumors. This phenotype is specifically driven by the deficiency in this Vav family member, because we have not observed statistically significant increases in deaths/tumor rates in *Vav2* ^–/–^, *Vav3* ^–/–^ or *Vav2* ^–/–^;*Vav3* ^–/–^ mice when compared to control littermates. The single *Rasgrf2* deficiency has only minor or no effects per se in promoting the widespread and thymus–localized subset of tumors, respectively. However, when combined with the inactivation of the *Vav1* gene, the *Rasgrf2* gene deficiency promotes a two–fold increase in the development of “widespread” lymphomas, shorter latency periods for the development of the disease, and enhanced mortality/sickness rates. These results suggest that the influence of the *Rasgrf2* gene loss on lymphomagenesis is totally contingent on the prior disruption of Vav1 function in the T–cell compartment. Despite the above data, it is worth noting that the tumoral phenotype described here requires long latency periods to develop, is not fully penetrant, and is similar anatomopathologically and immunophenotypically to the lymphomas spontaneously arising in wild type animals. These observations indicate that the *Vav1* and *Rasgrf2* gene deficiencies do not trigger single–handedly transformation but, rather, may offer an extra selective advantage for the tumorigenic events that trigger the fortuitous development of lymphomas in the B10.BR mouse strain used in this study.

What is the basis of this enhanced tumorigenesis? In the case of Vav1, the results reported here are totally at odds with the known functions of this protein in cell signaling, proliferation and oncogenesis [24]. Furthermore, Vav1 is also important in the T–cell compartment for the assembly of optimal signaling responses directly or indirectly related to cell growth, such as specific T–cell developmental transitions, thymocyte selection steps, and proliferative responses to antigens in the case of mature T–lymphocytes [25]. Such functions rely on the activation of Rac1, the Ras route and, in some cases, catalytically–independent functions [25], [26]. Given the implication of Vav1 in positive and negative TCR selection events in thymocytes, it could be speculated that the perturbation of thymic development or, alternatively, the lack of elimination of immature T–cells carrying tumorigenic rearrangements of antigen receptors in *Vav1* ^–/–^ mice could create a pool of tumorigenic events that may eventually increase lymphoma development rates in those animals. We do not support such possibility, since we are not aware of any report indicating that mouse strains with defective selection defects in the thymus are prone to tumorigenesis in the T–cell compartment. On the other hand, it could be also argued that the deficient signaling derived from the Vav1 deficiency will favor other signaling routes in specific thymocyte subsets or, alternatively, place an additional pressure on pre–neoplasic *Vav* ^–/–^ thymocytes that could create a Darwinian field to promote the selection of highly mitogenic thymocytes carrying pro–tumorigenic mutations in other signal transduction elements. In this context, it is worth mentioning that Tybulewicz’s group has shown recently that deficiencies in either Rac or Vav family genes may favor the survival of TCRβ^–^ thymocytes due to hyperactivation of the Notch signaling pathway [27], a route usually deregulated in human lymphoblastic leukemia [28]. Cantrell’s group has also shown that the elimination in thymocytes of RhoA function, a GTPase activated by Vav family proteins, leads to a lymphoblastic lymphoma very similar to the disseminated tumors found in *Vav1* ^–/–^ and *Vav1* ^–/–^;*Rasgrf2* ^–/–^ mice [29]. These data suggest that the role of Vav1 in thymic lymphomagenesis could be linked to dysfunctions in its direct downstream targets Rac1 and/or RhoA.

RasGRF2 promotes, similarly to Vav1, positive responses in mature T–cells [18]. However, unlike the case of Vav1, there is evidence indicating that it may also exert some tumor–suppressor activities. Thus, several reports have shown decreased expression and/or increased methylation of the *Rasgrf2* gene in cancer cell lines and primary tumors [12]-[15]. Moreover, it has been demonstrated that the overexpression of RasGRF2 negatively affects the transforming properties of a colon cancer cell line [15]. Despite these observations, we do not consider that the putative tumor suppressor activity of RasGRF2 is a plausible explanation for the results reported here, because the *Rasgrf2* deficiency only contributes to tumorigenesis when combined with the *Vav1* proto–oncogene loss. It is possible therefore that this cooperativity in lymphomagenesis promotion is just another reflection of the synergistic interactions occurring between the Vav1– and the RasGRF2–dependent pathways in normal T–cells. Hence, and similarly to the observations reported here, we have observed before that the endogenous RasGRF2 plays important, but subsidiary roles to the Vav1 route, in T–cell signaling. For example, the *Rasgrf2* gene deficiency does not affect the proliferation and blast formation of TCR–stimulated T–lymphocytes but, instead, it aggravates the already defective proliferative responses of Vav1–deficient T–cells to TCR–dependent signals [18]. Since the lack of CD69 expression in the *Vav1* ^–/–^;*Rasgrf2* ^–/–^ tumor cells suggests that their growth is mediated by TCR–independent mechanisms, the synergistic interaction between the *Vav1* and *Rasgrf2* gene deficiencies will probably need the alteration of TCR–independent routes rather than the canonical TCR downstream pathways for this hypothesis to be correct. The discrimination of all these regulatory possibilities will have wait to the identification of the signaling deficiency promoting the tumorigenesis in Vav1–deficient lymphocytes.

The observation that *Vav1* and *Rasgrf2* gene deficiencies promote enhanced tumorigenic rates in mice led us to verify whether the expression of these genes could be deregulated in human hematological tumors. Unfortunately, there are no many data available on T–cell lymphomas/leukemias, so we could not directly address changes in the expression of these two genes in these cancer subtypes. In order to get a general view of the expression of these two genes in hematological tumors, we have carried out expression studies by quantitative RT–PCR in a limited collection of hematopoietic tumors and, in addition, by in silico profiling using microarray data publicly available at the Oncomine database. The take home message of these studies is that the human *VAV1* and *RASGRF2* genes do not show a consistent and generalizable change pattern in hematopoietic tumors such as lymphomas, leukemias or myelomas. However, we did find small patient subgroups affected by some of those pathologies that show statistically significant upregulation/downmodulations of these two transcripts. Interestingly, we did not detect a coincidence in the change patterns of those two genes in the tumors analyzed so, if they contribute to the tumorigenesis, they must do so in cooperation with other mutations. It will be really important in the future to obtain a large group of T–cell lymphoma/leukemia samples in order to assess the specific implication of Vav1 and RasGRF2 in this tumorigenic context.

Not withstanding the actual role of these two proteins in T–cell lymphomas/leukemias, our observations do have some interest from a therapeutical point of view. Thus, it has been always assumed that the inactivation of the function of Vav and Rho/Rac family proteins could be of interest to treat cancer cells. The interest on this possibility has been also fueled by observations indicating that Vav and Rho/Rac family members are overexpressed in certain types of human tumors [2], [30]-[35]. However, our present data puts a note of caution on those possible avenues, since they indicate that the blockage of Vav family–dependent signaling could be highly counterproductive in the case of lymphoblastic lymphoma patients with reduced levels of *RASGRF2* gene expression in tumor cells. Hence, it will be important in the future to evaluate the beneficial/adverse effect of Vav family–directed therapeutics in different tumor types before initiating the search for Vav–specific drugs and clinical trials with human patients.

## Materials and Methods

### Mouse Strains

*Vav1^–/–^*, *Rasgrf2* ^–/–^, *Vav1* ^–/–^;*Rasgrf2* ^–/–^, *Vav2* ^–/–^, *Vav3* ^–/–^ and *Vav2* ^–/–^;*Vav3* ^–/–^ mice have been previously described [18], [36]-[38]. All mice used in these experiments were bred and maintained in the SPF Animal Facility of the University of Salamanca using 12–hour light/darkcycles. All animal–based experiments were approved by the Animal Use and Welfare Committees of the CSIC and University of Salamanca as well as by the Bioethics Comittee of the University of Salamanca. These approvals include review of the experimental protocols to be used in the animal work and, in addition, a description of the measures taken to avoid animal discomfort or pain.

### Histological and Immunohistochemical Analyses

Selected tissues were fixed in a buffered 4% formaldehyde solution for 48 h and paraffin–embedded. Sections were cut, stained with either hematoxylin/eosin (Sigma) and processed for immunohistochemistry using antibodies to mouse CD3 (clone F7.2.38, 1∶200 dilution, Dako) and PCNA (clone PC10, 1∶100 dilution, Cell Signaling). Immunoreactive signals were developed using horseradish peroxidase–conjugated secondary antibodies to mouse immunoglobulins (GE Healthcare).

### Flow Cytometry Analysis

Single cell suspensions were prepared by homogenization of tissues with the aid of 50 µm filters (Falcon). In the case of bone marrow cells, phosphate–buffered saline solution was flushed gently into the medullar cavity of isolated femoral bones and resulting cell suspensions washed using cycles of low–speed centrifugation and resuspension in phosphate–buffered saline solution. After erythrocyte lysis using 0.17 M NH_4_Cl, lymphocytes were stained with appropriate combinations of fluorescein–labeled anti–CD4, phycoerythrin–labeled anti–CD3, and allophycocyanin–labeled anti–CD8 antibodies. For the determination of thymic populations and splenic lymphocytes, cell suspensions were stained with antibodies to surface markers using appropriate combinations of fluorescein–labeled anti–CD4 and anti–CD44 antibodies, peridinin chlorophyll protein–labeled anti–CD4, anti–CD25 and anti–CD69 antibodies and allophycocyanin–labeled anti–CD4, anti–CD8 and anti–B220 antibodies. All the antibodies were obtained from BD Biosciences. Flow cytometry analyses were conducted using a FACSCalibur system (BD Biosciences) and analyzed using the Cell Quest (BD Biosciences), BD Paint–a–Gate (BD Biosciences), and WinMDI 2.8 softwares [18].

### Quantitative RT–PCR Analysis

A panel of cDNA samples obtained from normal and tumoral lymphoid samples (TissueScan Lymphoma Tissue qPCR Array I (2), Cat. Number LYRT101, Origene Technologies) was used to analyze the expression of *VAV1* and *RASGRF2* transcripts in different subsets of lymphomas. A detailed description of the lymphoma samples contained in that array can be obtained in the web of the commercial supplier (http://www.origene.com/geneexpression/disease-panels/products/LYRT101. aspx). Quantitative PCR reactions were performed in the IQ5 Real–Time PCR detection system (BioRad) using the SYBR^®^ Green^TM^ two–step qRT–PCR kit (Invitrogen). Sequences of the primers used for the amplification of the human *VAV1*, *RASGRF2* and *GAPDH* are available upon request. As comparative control, we used the average of the expression of those genes in six independent wild type lymphoid tissues (which was given an arbitrary value of 1). The expression values obtained in each experimental sample were also normalized taking into consideration the expression levels of the *GAPDH* gene. Quantitative PCR analyses were performed in three independent replicas that yielded similar results.

### In Silico Analysis of the Expression of VAV1 and RASGRF Family Members in Hematological Tumors

To carry out the comparative analysis between normal and tumor cells, we used the “differential cancer versus normal analysis” tool available at the Oncomine database (www.oncomine.org). The settings used for the searches were: gene: *VAV1* or *RASGRF2*; dataset size: microarray data containing a minimum of 75 independent samples; microarray platform: unrestricted; gene rank: top 10%; *P* value≤0.001. CODA analysis were done using the “outlier analysis” tool present in the Oncomine database. The settings for the searches were: gene: *VAV1* or *RASGRF2*; cancer type: leukemia, lymphoma and myeloma; dataset size: microarray data containing a minimum of 75 independent samples; microarray platform: unrestricted; gene rank: top 10%; *P* value≤1×10^–4^; COPA value: less than –3 or more than 3 for the downregulated and upregulated outliers, respectively.

### Statistical Analysis

We used the logrank/Mantel–Cox test for **Figure 1A**, the CODA algorithm for **Figure 6** and **Tables 2**–**3** and the Student’s *t*–test for the rest of analyses.
